# Dynamic Stiffness Analysis and Measurement of Radial Active Magnetic Bearing in Magnetically Suspended Molecular Pump

**DOI:** 10.1038/s41598-020-57523-8

**Published:** 2020-01-29

**Authors:** Jinji Sun, Han Zhou, Ziyan Ju

**Affiliations:** Beihang University, School of Instrumentation and Opto-electronics Engineering, Beijing, 100191 China

**Keywords:** Mechanical engineering, Electronics, photonics and device physics

## Abstract

Current stiffness and displacement stiffness are two important parameters of radial active magnetic bearing (RAMB) that are generally considered as constants in a control system. However, such presumption may lead to the probable degradation of the control performance of the RAMB system when the perspective that the current and displacement stiffness should be variable due to variations in the speed. On this regard, a structure analysis and stiffness measurement method based on non-variable stiffness would not be feasible for RAMB under high-speed conditions. This paper presented an analysis of the dynamic stiffness characteristics of RAMB by means of a dynamic equivalent magnetic circuit method and laid out a comparison between the results of the theoretical analysis and the simulation results of finite element method. In particular, a novel dynamic stiffness measurement method of RAMB under high-rotation frequency was introduced. Results of experiments on the dynamic stiffness of RAMB demonstrated an excellent agreement with the theoretical research and the finite element analysis results, thereby verifying the rationality of the discussed dynamic stiffness characteristics. Practically, the proposed measurement method of RAMB dynamic stiffness provides an accurate analysis for the dynamic stiffness and contributes inspiring research significance for the dynamic properties of RAMB.

## Introduction

A molecular pump is kind of high-end scientific instrument, such as cyclotron, laser, mass spectrometer, gyro equipment and so on, typically used in obtaining high vacuum^1,2^. Bearings are used in molecular pumps to reduce friction and oil contamination. Compared to the traditional ball bearings and oil film bearings, the magnetic bearing is a new type of bearing with special advantages of non-contact characteristics, much less friction, low power consumption, low maintenance cost, dynamical controllability and active control ability of rotor dynamic imbalance, giving it broad prospects in industrial applications^3–6^. The magnetic bearing used in molecular pumps can realise oil-free, wear-free and quiet operation and minimal vibration, which are especially suitable conditions for the semiconductor industry, such as in ultra-high vacuum applications^7–9^.

Accordingly, the two most commonly used magnetic bearing types are the passive and active magnetic bearings. On one hand, passive magnetic bearings with low power consumption realise the passive suspension of rotor through the interaction between the stator and rotor permanent magnets or the reluctance suction between the stator and rotor^10–12^. However, these bearings lack active controllability and exhibit low damping and stiffness. On the other hand, active magnetic bearings, which generate supporting force by means of electromagnets, a feedback control loop and other elements as sensors and power amplifiers, present extraordinary characteristics in terms of high stiffness, good controllability and damping properties relative to the passive ones^13–16^. Consequently, most magnetically suspended molecular pumps adopt active magnetic bearings to achieve ultra-high pumping speed at present.

Stiffness is generally known to be one of the most important parameters for the whole control system^17,18^ of magnetic bearings and is categorised mainly into displacement stiffness and current stiffness. Literature^19^ asserted that both coefficients of current and displacement stiffness for a power magnetic bearing can be precisely determined via application of an open-loop static levitation force on the rotor. Reference^20^ put forward a new stiffness measurement method of repulsive passive axial magnetic bearing with Halbach magnetic array, for the single gimbal magnetically suspended control moment gyroscope. Moreover, literature^21^ proposed a new stiffness measurement method for the magnetically suspended flywheel for determining the current stiffness and displacement stiffness of permanent magnet biased radial magnetic bearings. Further, a detailed stiffness measurement method for a radial hybrid magnetic bearing was described in^22^. In all these studies, the stiffness of magnetic bearings has been regarded as a constant (not affected by rotation frequency) and the measurement experiments were performed when the rotor was suspended steadily at zero-frequency. For the low speed applications in^20–22^, the stiffness at zero speed can be used as a constant in the whole speed range. However, some researchers have found that the stiffness of radial active magnetic bearing (RAMB) would decline in a discernible manner with an increase in rotor frequency and would influence the stability of the radial magnetic bearing system in the process^23,24^. The literature presented herein also depict that the frequency response of the current stiffness could be obtained by addition of a balancing mass; nonetheless, the method is complicated and no explicit description of measuring current stiffness is provided in this research. This aside, the frequency response of the displacement stiffness is not analysed nor measured. Therefore, it is necessary implement the meterage of dynamic stiffness of RAMB for the magnetically suspended molecular pump (MSMP).

In this paper, an accurate dynamic stiffness measurement method of RAMB is put forward for MSMP. A build-up of the characteristics of the RAMB dynamic stiffness is first established by means of an equivalent magnetic circuit with varying frequency. Diagrams of magnetic line distribution and change trend of the stiffness is then obtained using finite element method (FEM). Finally, the proposed dynamic stiffness measurement method of RAMB is verified by prototyped experiments via comparison of results of the theoretical and FEM analyses.

## Results

### Analysis of dynamic stiffness characteristics of RAMB for MSMP

**Figure 1:**
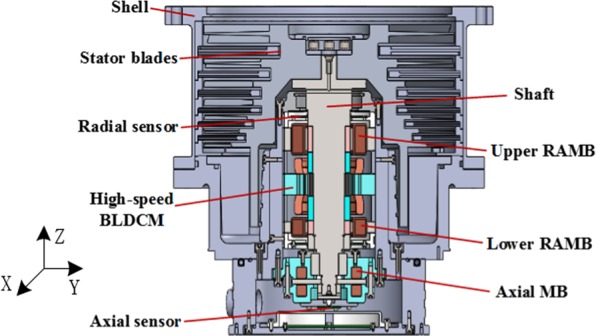
A magnetically suspended molecular pump model.

**Figure 2:**
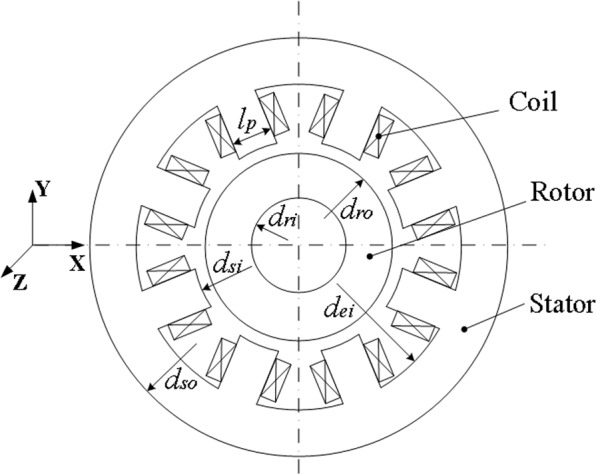
Design configuration of RAMB.

Figure 1 shows the magnetically suspended molecular pump model, mainly composed of a single rotor shaft, two RAMBs, an axial active magnetic bearing, a high-speed brushless direct current motor, two integrated displacement sensors and stator blades. For the high pumping-speed MSMP CXF400–4000, designed by professors and doctors in our academy and applied in civilian equipment, the configuration of the RAMB is pictured in Fig. 2. In the figure, the materials of the stator and rotor core are silicon steels with corresponding dimensions as described in Table 1.

**Table 1:** Key dimensions of RAMB.

Dimension	Value
Stator outer diameter (*d*_*so*_)	72 mm
Pole axial length of stator (*l*_*a*_)	13.5 mm
Stator yoke inner diameter (*d*_*ei*_)	64 mm
Stator inner diameter (*d*_*si*_)	34.6 mm
Rotor outer diameter (*d*_*ro*_)	34 mm
Rotor inner diameter (*d*_*ri*_)	26 mm
Half angle between two adjacent poles (*α*)	22.5°
Pole width of stator (*l*_*p*_)	5.5 mm
Turn number of coils (*N*)	300 turns/pole
Air gap length (*g*)	0.3 mm
Laminated thickness (*d*)	0.35 mm
Conductivity (*σ*)	7.46 × 10^6^ S/m
Relative magnetic permeability (*μ*_*r*_)	5000

While the rotor operates at or near the rated speed, eddy currents caused by time-varying control currents will affect the resistance of the rotor and the stator, which is an important factor influencing the stiffness^23^. Such phenomenon results in substantial reduction in stiffness compared to the 0 Hz stiffness value that is considered constant in the RAMB system. As such, the traditional magnetic circuit model, which ignores the rotor frequency variation and defaults the control current and magnetic resistances to a constant value, is not suitable for the RAMB stiffness analysis. Through an analysis of the dynamic characteristics when the rotor speed is variable while other parameters remain unchanged, studies in^23,24^ have proved that rotor frequency variation and eddy current effect leave a marked impact on magnetic bearing stiffness. Accordingly, these studies established an analytical method for a hybrid radial magnetic bearing system based on an equivalent circuit model whose parameters were frequency-dependent. Although it has been generally recognised that eddy current effects could be mitigated by replacing the solid structure with laminated cores and by designing modifications, the magnetic bearings stiffness was still considerably influenced by eddy currents when the rotation frequency was sufficiently high^25–27^. Therefore, it is necessary and is of great significance to carry out a quantitative analysis on the dynamic stiffness of RAMB at different rotation speeds.

Specifically, the rotor speed change will lead to a time-varying control current, which will induce the eddy current effect in the stator core and rotor core, leading to the modification of their magnetic permeability. Based on the dynamic permeability of the stator and rotor, an analysis of accurate quantitative dynamic stiffness characteristics for the varying frequencies can be performed by establishment of an equivalent magnetic circuit.

The relative permeability of stator and rotor with different rotation frequencies has been analysed in^25^. In particular, the relative permeability of stator (*μ*_*rs*_) could be deduced using Eq. (1) while the relative permeability for rotor could be determined by Fourier transform and the n-th relative permeability (*μ*_*nr*_) can be expressed through Eq. (2). Here, *k*_*n*_ is a coefficient determined by the stator structure; the other parameters are assigned in Table 1,

1 $${\mu }_{rs}(f)={\mu }_{r}\frac{\tanh \left(\sqrt{jf\sigma {\mu }_{0}{\mu }_{r}}\frac{d}{2}\right)}{\sqrt{jf\sigma {\mu }_{0}{\mu }_{r}}\frac{d}{2}};$$

2 $${\mu }_{nr}(f)={\mu }_{r}\frac{\tanh \left(\sqrt{jf\sigma {\mu }_{0}{\mu }_{r}{k}_{n}{r}_{ro}+jf\sigma {\mu }_{0}{\mu }_{r}}\frac{d}{2}\right)}{\sqrt{jf\sigma {\mu }_{0}{\mu }_{r}{k}_{n}{r}_{ro}+jf\sigma {\mu }_{0}{\mu }_{r}}\frac{d}{2}}.$$

**Figure 3:**
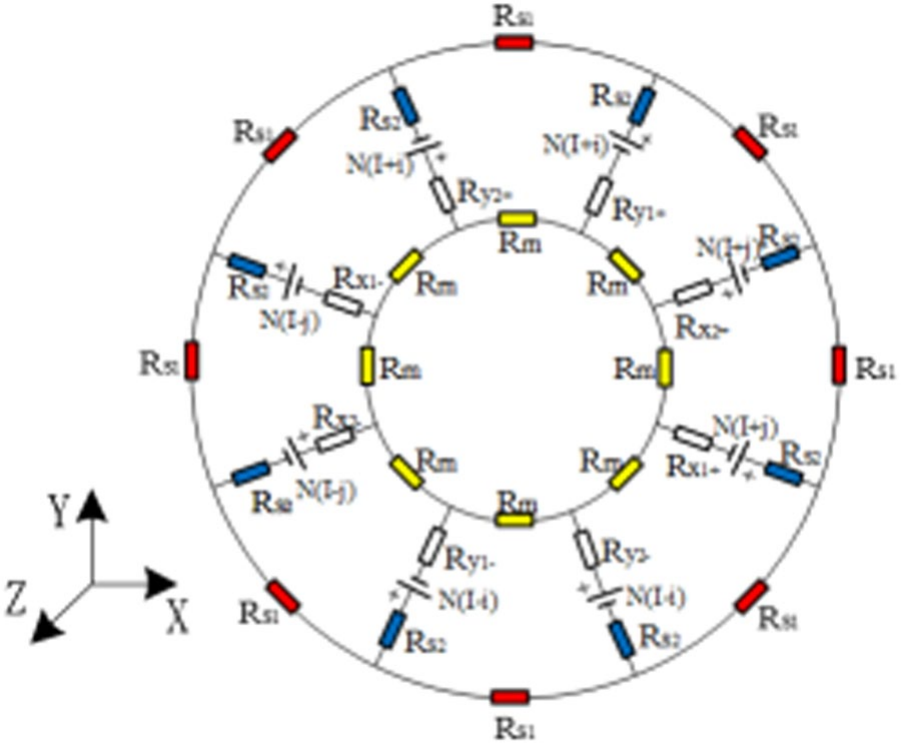
Dynamic equivalent magnetic circuit model of RAMB.

Figure 3 shows the dynamic equivalent magnetic circuit model of RAMB, in which *R*_*s1*_ and *R*_*s2*_ are the equivalent magnetic reluctances of the stator; *R*_*x1+*_, *R*_*x2+*_, *R*_*x1−*_, *R*_*x2−*_, *R*_*y1+*_, *R*_*y2+*_, *R*_*y1−*_ and *R*_*y2−*_ are utilised to depict the magnetic reluctances of the air gap in *x*_*1+*_, *x*_*2+*_, *x*_*1−*_, *x*_*2−*_, *y*_*1+*_, *y*_*2+*_, *y*_*1−*_ and *y*_*2−*_ direction, respectively and; *R*_*rn*_ is the dynamic reluctances of the *n-th* harmonic of the rotor. According to the distribution of magnetic flux density, whereas the rotor is in the central position, the reluctances can be calculated based on Eqs. (1) and (2) as follows:

3 $${R}_{x1+}={R}_{x2+}={R}_{x1-}={R}_{x2-}={R}_{y1+}={R}_{y2+}={R}_{y1-}={R}_{y2-}=R=\frac{g}{{\mu }_{0}{l}_{a}{l}_{p}},$$

4 $${R}_{s1}(f)=\frac{\pi ({r}_{so}+{r}_{ei})\sqrt{if\sigma {\mu }_{0}{\mu }_{r}}\frac{d}{2}}{8{\mu }_{0}{\mu }_{r}{l}_{a}({r}_{so}-{r}_{ei})\tanh \left(\frac{d}{2}\sqrt{if\sigma {\mu }_{0}{\mu }_{r}}\right)},$$

5 $${R}_{s2}(f)=\frac{d({r}_{so}+{r}_{ei}-2{r}_{si})\sqrt{if\sigma {\mu }_{0}{\mu }_{r}}}{4{\mu }_{0}{\mu }_{r}{l}_{a}{l}_{p}\,\tanh \left(\frac{d}{2}\sqrt{if\sigma {\mu }_{0}{\mu }_{r}}\right)},$$

6 $${R}_{rn}(f)=\frac{\pi ({r}_{so}+{r}_{ei})\sqrt{if\sigma {\mu }_{0}{\mu }_{r}(1+n)}\frac{d}{2}}{8{\mu }_{0}{\mu }_{r}{l}_{a}({r}_{so}-{r}_{ei})\tanh \left(\frac{d}{2}\sqrt{if\sigma {\mu }_{0}{\mu }_{r}(1+n)}\right)}.$$

#### Model of dynamic current and displacement stiffness

Dynamic current stiffness. Neglecting the coupling between two directions *x* and *y*, as the rotor rotates at the balance location, the magnetic fluxes in the *x*+ and *x*− directions could be denoted as *ϕ*_*x+*_ and *ϕ*_*kx−*_, as depicted based on Ohm’s law of magnetic resistance as

7 $${\phi }_{x+}(f)=\frac{2N(I+i)}{\rho {R}_{tx+}(f)},$$

8 $${\phi }_{x-}(f)=\frac{2N(I-i)}{\rho {R}_{tx-}(f)}$$

where *ρ* is the magnetic flux leakage coefficient; *I* is the bias current; *i* is the control current and; *R*_*tx+*_ and *R*_*tx−*_ are respectively, the total reluctance of the magnetic circuit in the *x*+ and *x*− directions. According to the formula for calculating electromagnetic force, the magnitude of the electromagnetic force in the *x*+ and *x*− directions could be respectively expressed as:

9 $${f}_{x+}(f)=\frac{{\phi }_{x+}^{2}(f)}{2{\mu }_{0}\frac{{l}_{a}{l}_{p}}{\cos (\alpha )}}=\frac{8{N}^{2}{(I+i)}^{2}}{{\rho }^{2}{\mu }_{0}{l}_{a}{l}_{p}}\cdot \frac{1}{{R}_{tx+}^{2}(f)}\cdot \,\cos (\alpha ),$$

10 $${f}_{x-}(f)=\frac{{\phi }_{x-}^{2}(f)}{2{\mu }_{0}\frac{{l}_{a}{l}_{p}}{\cos (\alpha )}}=\frac{8{N}^{2}{(I-i)}^{2}}{{\rho }^{2}{\mu }_{0}{l}_{a}{l}_{p}}\cdot \frac{1}{{R}_{tx-}^{2}(f)}\cdot \,\cos (\alpha ).$$

In the differential mode, the force in the *x*-direction of the rotor represents the difference between the electromagnetic forces in the *x*+ and *x*− directions. The magnitude is denoted as *f*_*x*_ and the calculation equation is

11 $${f}_{x}(f)={f}_{x+}(f)-{f}_{x-}(f)=\frac{8{N}^{2}\cdot \,\cos (\alpha )}{{\rho }^{2}{\mu }_{0}{\mu }_{r}{l}_{a}{l}_{p}}\cdot \left[\begin{array}{c}(\frac{2I}{{R}_{tx+}^{2}(f)}+\frac{2I}{{R}_{tx-}^{2}(f)})i+(\frac{1}{{R}_{tx+}^{2}(f)}-\frac{1}{{R}_{tx-}^{2}(f)}){i}^{2}\\ \,+\,(\frac{1}{{R}_{tx+}^{2}(f)}-\frac{1}{{R}_{tx-}^{2}(f)}){I}^{2}\end{array}\right].$$

When the rotor is suspended at the equilibrium position, ${R}_{tx+}^{2}={R}_{ty-}^{2}={R}_{t}^{AC}$, *i*^2^ can be ignored due to the fact that the control current *i* is a tiny value compared to the bias current *I*. Here, the dynamic current stiffness *k*_*i*_ can be defined as

12 $${k}_{i}(f)=\frac{d{f}_{x}(f)}{di}=\frac{32{N}^{2}I}{{\rho }^{2}{\mu }_{0}{l}_{a}{l}_{p}}\cdot \,\cos (\alpha )\cdot {\left[\begin{array}{c}\frac{2g}{{\mu }_{0}{l}_{a}{l}_{p}}+\frac{\pi ({r}_{so}+{r}_{ei})\sqrt{if\sigma {\mu }_{0}{\mu }_{r}}\frac{d}{2}}{8{\mu }_{0}{\mu }_{r}{l}_{a}({r}_{so}-{r}_{ei})\tanh (\sqrt{if\sigma {\mu }_{0}{\mu }_{r}}\frac{d}{2})}+\frac{d\pi ({r}_{so}+{r}_{ei}-2{r}_{si})\sqrt{if\sigma {\mu }_{0}{\mu }_{r}}}{2{\mu }_{0}{\mu }_{r}{l}_{a}{l}_{p}\tanh (\sqrt{if\sigma {\mu }_{0}{\mu }_{r}}\frac{d}{2})}\\ +\frac{\pi ({r}_{so}+{r}_{ei})\sqrt{2if\sigma {\mu }_{0}{\mu }_{r}}\frac{d}{2}}{8{\mu }_{0}{\mu }_{r}{l}_{a}({r}_{so}-{r}_{ei})\tanh (\frac{d}{2}\sqrt{2if\sigma {\mu }_{0}{\mu }_{r}})}\end{array}\right]}^{-2}.$$

**Figure 4:**
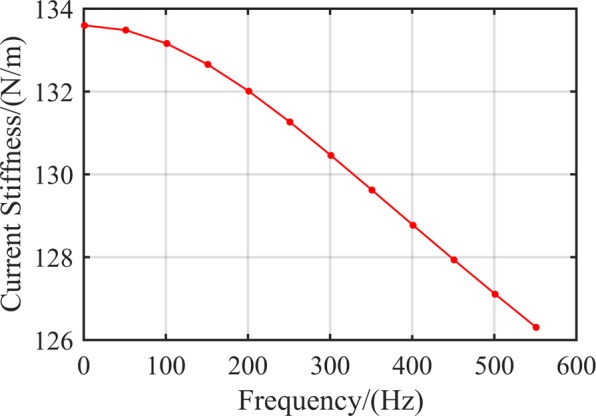
Dynamic current stiffness *k*_*i*_ at different frequencies.

Figure 4 depicts the relationship between RAMB current stiffness and rotor frequency based on the dimensions listed in Table 1. Note that the value of current stiffness declined with an increase in rotor frequency. Compared at 0 Hz, the current stiffness at 500 Hz could be seen to have decreased by 4.9%. Such distinct changes could be expected to inevitably affect the dynamic performance of RAMB.

Dynamic displacement stiffness. Let *d*_*x*_ denote the displacement of the rotor in the *x*-direction that is much smaller than the length of air gap *g*. The expressions of air gap reluctance in *x*+ and *x−* directions can be expressed as

13 $${R}_{gx1+}={R}_{gx2+}=\frac{g-dx\,\cos (22.5^\circ )}{{\mu }_{0}{l}_{a}{l}_{p}},$$

14 $${R}_{gx1-}={R}_{gx2-}=\frac{g+dx\,\cos (22.5^\circ )}{{\mu }_{0}{l}_{a}{l}_{p}}.$$

In a similar manner as Eqs. (7) through (12), the RAMB electromagnetic force can be calculated with Eqs. (15) through (17); consequently, the dynamic displacement stiffness when the higher order of *d*_*x*_ is ignored can be obtained from Eq. (18):

15 $${f}_{x+}(f)=\frac{{\phi }_{x+}^{2}(f)}{2{\mu }_{0}{l}_{a}{l}_{p}}=\frac{8{N}^{2}{(I+i)}^{2}}{{\rho }^{2}{\mu }_{0}{l}_{a}{l}_{p}}\cdot \frac{1}{{R}_{tx+}^{2}(f)}\cdot \,\cos (\alpha ),$$

16 $${f}_{x-}(f)=\frac{{\phi }_{x-}^{2}(f)}{2{\mu }_{0}{l}_{a}{l}_{p}}=\frac{8{N}^{2}{(I+i)}^{2}}{{\rho }^{2}{\mu }_{0}{l}_{a}{l}_{p}}\cdot \frac{1}{{R}_{tx-}^{2}(f)}\cdot \,\cos (\alpha ),$$

17 $${f}_{x}(f)={f}_{x+}(f)-{f}_{x-}(f)=\frac{8{N}^{2}{(I+i)}^{2}}{{\rho }^{2}{\mu }_{0}{l}_{a}{l}_{p}}\cdot \,\cos (\alpha )\cdot \left(\frac{1}{{R}_{tx+}^{2}(f)}-\frac{1}{{R}_{tx-}^{2}(f)}\right),$$

18 $${k}_{s}(f)=\frac{64{N}^{2}{(I+i)}^{2}{\cos }^{2}(\alpha )}{{\rho }^{2}{\mu }_{0}^{2}{l}_{a}^{2}{l}_{p}^{2}}\cdot {\left[\begin{array}{c}\frac{\pi ({r}_{ro}+{r}_{ei})}{8{\mu }_{0}{\mu }_{r}{l}_{a}({r}_{ro}-{r}_{ei})}+\frac{{r}_{so}+{r}_{ei}-2{r}_{si}}{{\mu }_{0}{\mu }_{r}{l}_{a}{l}_{p}}\\ +\frac{\pi d({r}_{ro}+{r}_{ri})\sqrt{if\sigma {\mu }_{0}{\mu }_{r}}}{8{\mu }_{0}{\mu }_{r}{l}_{a}({r}_{ro}-{r}_{ri})\tanh (d\sqrt{if\sigma {\mu }_{0}{\mu }_{r}})}+\frac{2g}{{\mu }_{0}{l}_{a}{l}_{p}}\end{array}\right]}^{-3}.$$

**Figure 5:**
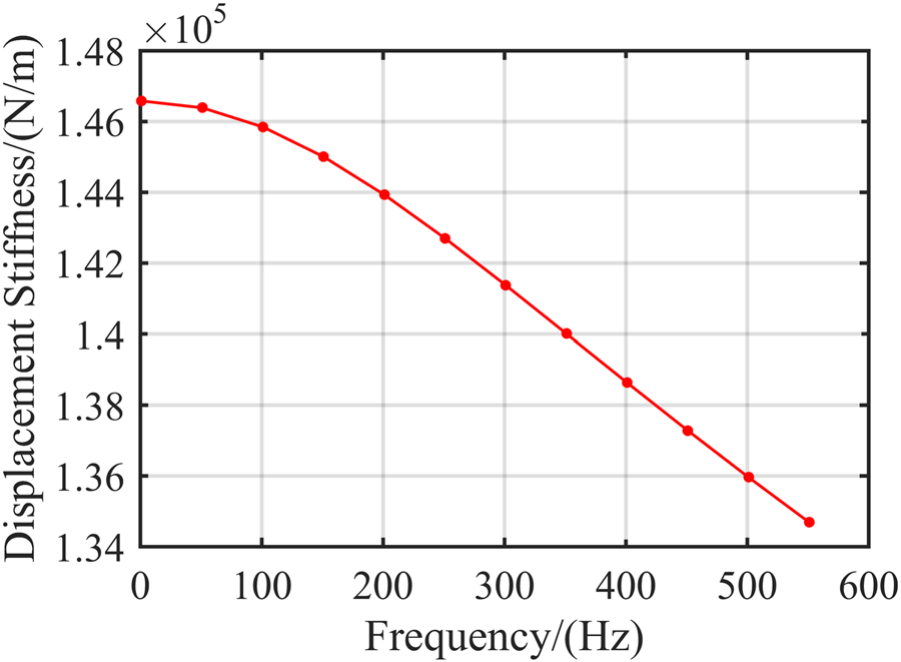
Dynamic displacement stiffness *k*_*x*_ at different frequencies.

Figure 5 illustrates the relationship between RAMB dynamic displacement stiffness and frequency, which demonstrated, in particular, that the variation tendencies of displacement stiffness and current stiffness are consistent with an increase in frequency.

#### Influences of control parameters and structural parameters on stiffness

For the RAMB structure shown in Fig. 2, the equation of electromagnetic force in RAMB can be linearised at the magnetic centre as^22^

19 $$f(x,i)={k}_{s}x+{k}_{i}i,$$

with *f* as a representation of the linear function of current and displacement and *k*_*s*_ and *k*_*i*_ being the respective displacement stiffness and current stiffness of magnetic bearings that are closely related to magnetic circuit characteristics and structural parameters. On such basis, the stiffness of the RAMB system can be obtained using Eq. (20), which defines it as the ratio of the electromagnetic force *f* and the rotor displacement that is dependent on current stiffness, displacement stiffness and the current. Furthermore, the current is a function of rotor displacement, which is dependent on the controller design of the RAMB system. Accordingly, the control and structure parameters have great influences on the system stiffness and need to be analysed.

20 $$K=\frac{df(x,i)}{dx}=\frac{\partial f(x,i)}{\partial x}+\frac{\partial f(x,i)}{\partial i}\cdot \frac{di}{dx}={k}_{{\rm{s}}}+{k}_{i}\frac{di}{dx}.$$

Stiffness analysis with different control parameters. When the rotor is acted upon by an external force, the result of force analysis in the *x*-direction can be obtained with Eq. (21). Essentially, Eq. (21) employs the Laplace transform, whereas Eq. (22) defines the transfer function with the external force as input and displacement as output:

21 $$m\ddot{x}=F-({k}_{i}i+{k}_{s}x),$$

22 $$\frac{X(s)}{F(s)}=\frac{1}{m{s}^{2}+{k}_{s}+{k}_{i}G(s)},$$

with *G*(*s*) as the total transfer function of the displacement sensor, PID controller and power amplifier. According to the theory of vibration, the equation of motion for second-order system can be expressed by Eq. (23), where *D* represents damping and *K* the stiffness of the RAMB system. Applying Eqs. (22) and (23), *s* = *2πfj* could be set and an equal stiffness of the RAMB system can be obtained through Eq. (24), with *ϕ*(*2πf*) for the phase angle of the transfer function *G*(*s*),

23 $$m\ddot{x}+D\dot{x}+K=F,$$

24 $${K}_{{\rm{e}}}(2\pi jf)={k}_{s}(2\pi f)+{k}_{i}(2\pi f)|G(2\pi jf)|\cos (\phi (2\pi f)).$$

The substantial influence of the control parameters on the equal stiffness for RAMB system has been clarified; moreover, the incomplete differential PID controller^28^, whose transfer function is presented as Eq. (25), is generally utilised to overcome high-frequency noises:

25 $${G}_{c}(s)={K}_{p}+{K}_{i}\frac{1}{s}+\frac{{K}_{d}s}{1+{T}_{d}s},$$

where *K*_*p*_, *K*_*i*_, *T*_*d*_ and *K*_*d*_ are the typical parameters of the incomplete differential PID controller, and *K*_*i*_ is finally designed according to the general actual effect for its decisive role in controlling the steady-state error. *K*_*p*_, *K*_*d*_ and *T*_*d*_, thus, become the main research objects in this part, whereas the other parameters such as the sample AD converter and current controller amplification coefficients are kept stable, as listed in Table 2.

**Table 2:** Main Coefficients of AD converter and Power amplifier.

Coefficient	Figure	Coefficient	Figure
Coil inductance (*L*)	540 mH	Coil resistance (*R*)	7.5 Ω
Power amplifier coefficient (*k*_*m*_)	0.75	AD converter coefficient (*k*_*a*_)	4096/3
Current controller coefficient (*k*_*ico*_)	5	Rotor mass (m)	1500 g

**Figure 6:**
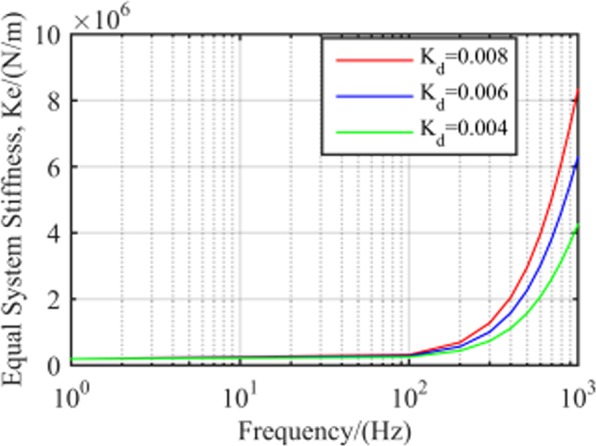
RAMB system equal stiffness with different *K*_*d*_.

Equal system stiffness with different *K*_*d*_. Figure 6 shows the stiffness characteristics of the close loop system for RAMB with different *K*_*d*_ values. Apparently, there was almost no difference in the equal stiffness of the system at the low frequency bands. However, in the medium- and high-frequency areas, the equivalent stiffness not only rose with higher frequency, but the magnitudes of the growth increased with *K*_*d*_ as well. Take for instance the case where *K*_*d*_ was set to 0.004, 0.006 and 0.008. Here, the increased equal stiffness from 400 to 1000 Hz were 3.1 × 10^6^, 4.8 × 10^6^ and 6.3 × 10^6^ N/m, respectively.

**Figure 7:**
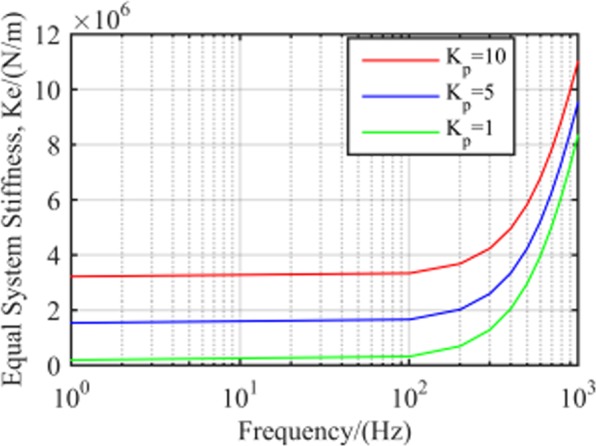
RAMB system equal stiffness with different *K*_*p*_.

Equal system stiffness with different *K*_*p*_. Figure 7 displays equal stiffness for the close loop system of RAMB with different *K*_*p*_ values. In this system, the equal stiffness would remain stable and the equivalent stiffness would be proportional to *K*_*p*_ at the low-frequency band. However, the equivalent stiffness increased with frequency in the medium- and high-frequency areas, and their differences as a result of different *K*_*p*_ values were invariable. For instance, the respective differences for *K*_*p*_ from 1 and between 5 and 10 were 1.2 × 10^6^ and 1.6 × 10^6^ N/m.

**Figure 8:**
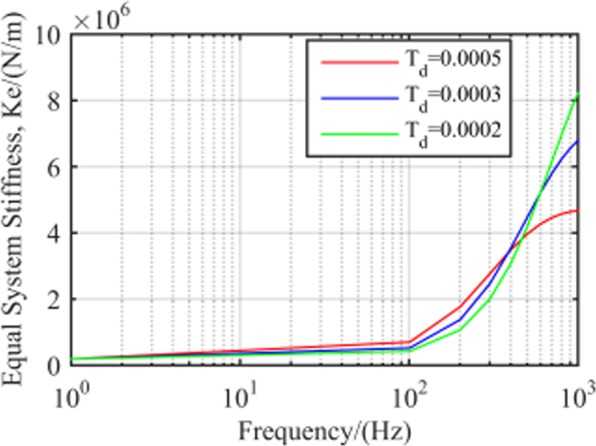
RAMB system equal stiffness with different *T*_*d*_.

Equal system stiffness with different *T*_*d*_. Figure 8 shows the stiffness characteristics of the RAMB system for different values of *T*_*d*_. The almost coincided and horizontal curves in the low-frequency band indicated that the system stiffness was not affected by the frequency and *T*_*d*_ value. However, in the medium- and high-frequency areas, the equivalent stiffness became higher with an increase in frequency, although the magnitude of such growth increased with a decline in *T*_*d*_. For example, setting *T*_*d*_ to 0.0002, 0.0003 and 0.0005 would increase equal stiffness from 400 to 1000 Hz at 5.1 × 10^6^, 3.3 × 10^6^ and 1.2 × 10^6^ N/m, respectively.

Stiffness analysis with different structural parameters. Equations (12) and (18) clearly depict that the values of current and displacement stiffness are influenced by many structural coefficients such as air gap length, pole width of the stator and coil turns. On this regard, this study carried out an analysis of the influence of structural parameters on dynamic stiffness.

**Figure 9:**
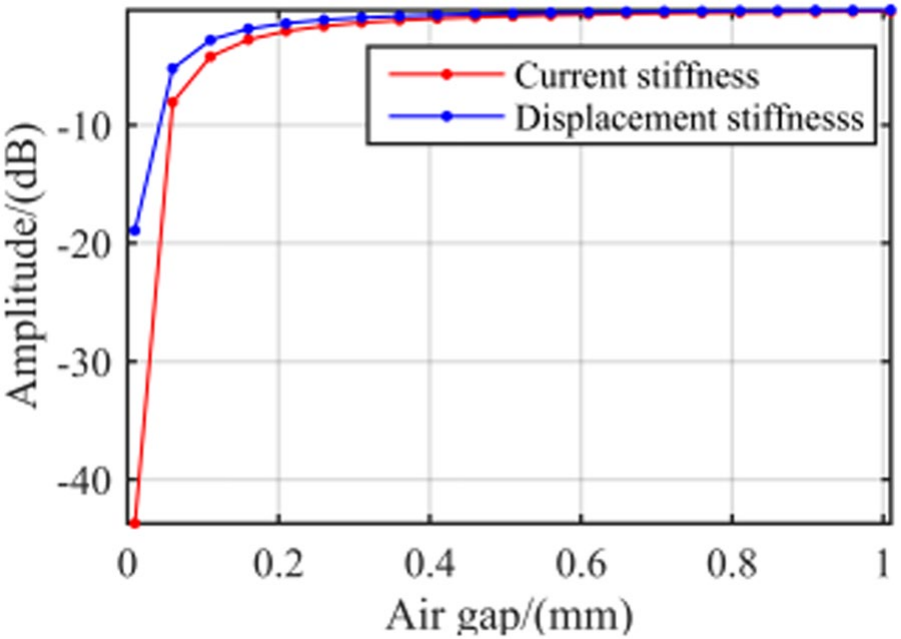
RAMB system dynamic stiffness with different air gap values.

Stiffness with air gap length. The variation of air gap length leaves a considerable influence on the magnetic reluctance of the air gap, leading to a corresponding variation in current and displacement stiffness of magnetic bearings. Herein, several air gap values were selected from 0 to 1 mm, for an investigation of the change of current and displacement stiffness at 1000 Hz. Figure 9 presents the results. The figure validated that current and displacement stiffness increase with air gap. Moreover, the rate of stiffness growth slowed down until zero as the air gap increased. Considering such decrease of magnetic force being directly caused by a large air gap, value of the air gap should not be too large; the 0.2–0.4-mm range would be perfect for the RAMB discussed in this paper.

**Figure 10:**
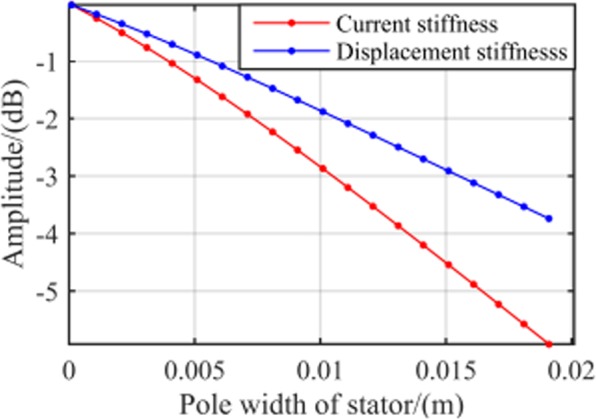
RAMB system dynamic stiffness with different pole width of stator.

Stiffness with pole width of stator. From the dynamic stiffness characteristics obtained in (12) and (18), it can be seen that the pole width of stator is also the key factor affecting the dynamic stiffness of magnetic bearing. Adopting the same analytical method as air gap length and the results are shown in Fig. 10. The analytical results indicate that the dynamic stiffness amplitudes decrease with the rising pole arc width of stator, thus the pole arc width should be chosen relatively small.

**Figure 11:**
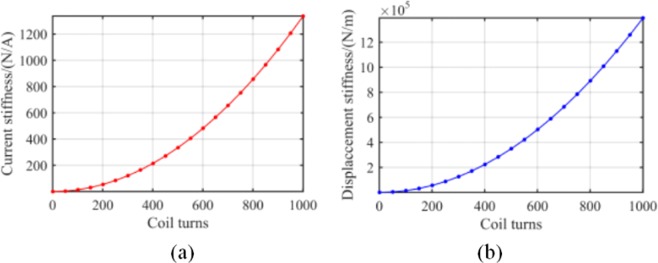
RAMB system dynamic stiffness with different coil turns: (**a**) current stiffness (**b**) displacement stiffness.

Stiffness with pole coil turns. As an important parameter of the proposed dynamic stiffness model shown in Eqs. (12) and (18), the value of coil turns has substantial impact on current and displacement stiffness. To study the change of dynamic stiffness with varying coil turns, several coil turn values were selected from 0 to 1000. Results of the analysis are presented in Fig. 11. In the figure, the exponentially rising curves signify that the number of turns of the coil should be set to a large value. However, it was necessary to consider that the increase of coil turns would lead to large inductance, which reduces the stability of the system in the actual RAMB design. Thus, a value from 200 to 400 is ideal for the RAMB discussed in this paper.

#### Finite element analysis of dynamic stiffness

**Figure 12:**
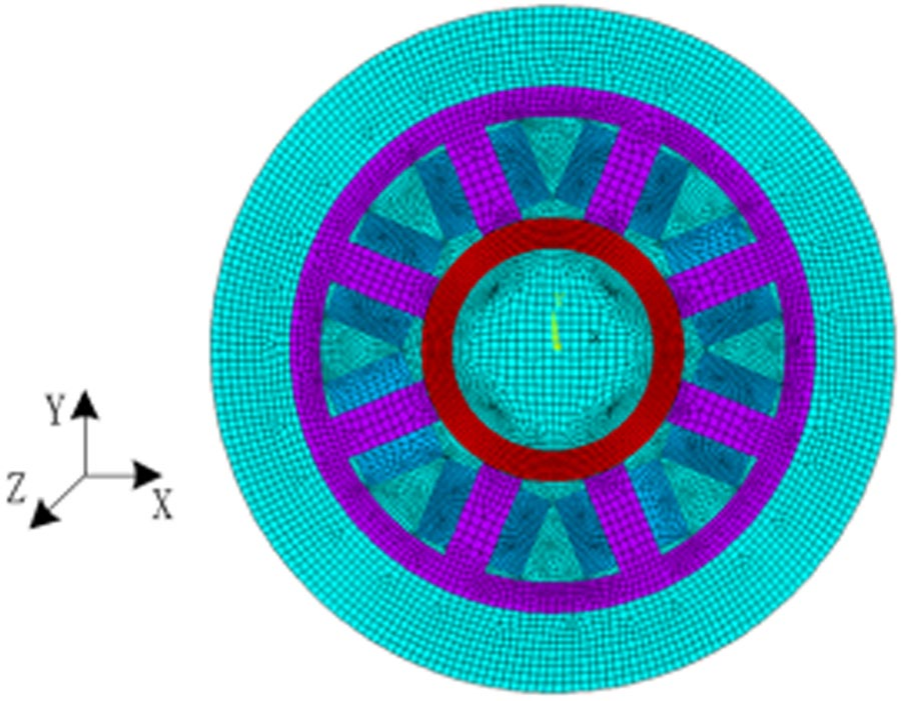
Finite element model of radial electromagnetic bias magnetic bearing.

**Figure 13:**
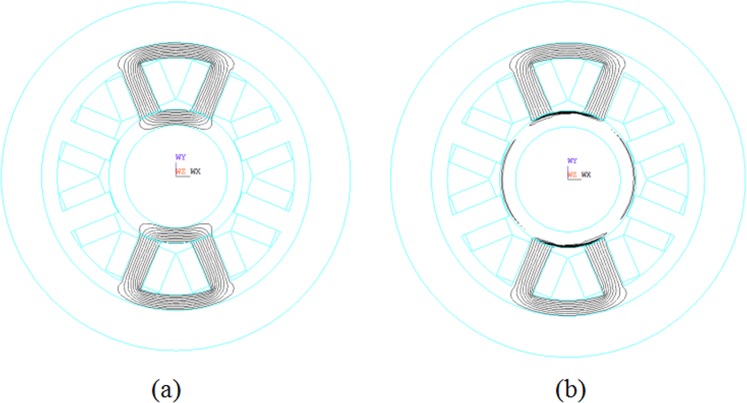
Magnetic induction line distribution at (**a**) 0 and (**b**) 800 Hz.

By the electromagnetic field analysis model in ANSYS software, the 2D-FEM mesh model of RAMB introduced in Fig. 2 was obtained as Fig. 12. For this model, the plane53 magnetic structure unit was used for its perfect performance of transient magnetic field analysis. Additionally, the rotor part was meshed by the mapped grid mode while the remaining part was meshed by the free mode. In particular, areas of air gap between the rotor surface and the fixed rotor where the electromagnetic field varies on a great scale, were finely meshed. In the FEM simulations, the bias current was set at 0.38 A, which is expected to generate a corresponding bias magnetic flux density, while the different rotor frequency values from 0 to 800 Hz were set for the rotor in the ANSYS software. Subsequently, a FEM simulation was carried out for each speed while the distributional patterns of the magnetic line of the magnetic bearing presented in Fig. 13 was obtained via the finite element calculation.

Based on Fig. 13(a), the magnetic lines of force were uniformly distributed when rotor was at a standstill. However, when the rotor rotated, the generated alternating magnetic field induced an alternating electric field that generated eddy currents and additional magnetic fields, thereby weakening the main magnetic field. As the rotor frequency increased, the theoretical analysis in^26^ indicated that the rotor magnetic induction line would no longer be evenly distributed but would tend to the rotor surface due to the skin effect caused by the eddy currents. For the RAMB structure and FEM simulation conditions described in this paper, the FEM simulation results were consistent with the theoretical analysis as depicted in Fig. 13(b), in which the flux lines distorted severely and were squeezed to the surface of the rotor. This indicates that the magnetic forces would decrease rapidly and that such phenomenon could be hardly observed in AMB rotor experiments at a rotation speed of 800 Hz. To demonstrate the decrease in the variation of stiffness and magnetic force for high rotor frequency, the RAMB experiments were performed in such a way that would ensure that the magnetic force remains unchanged and the control current adjusts adaptively with a change in rotor speed. The experimental results showed that the control current would significantly rise with higher rotation frequency, which can indirectly illustrate the sharp drop of the magnetic force for RAMB.

**Figure 14:**
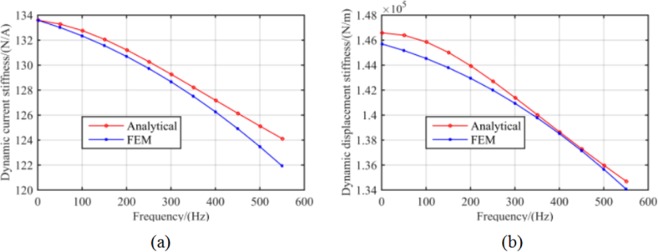
Amplitude of (**a**) dynamic current stiffness vs. rotor frequency and (**b**) dynamic displacement stiffness vs. rotor frequency.

Accordingly, the corresponding values of dynamic stiffness with varying rotor frequencies were calculated herein using 2D FEM and the calculated values were compared with those of the analytical results, as shown in Fig. 14. Here, note that the variation trends of dynamic stiffness were consistent with the theoretical analysis results. The maximum errors for current and displacement stiffness were 1.6% and 0.9%, respectively.

### Dynamic stiffness measurement method

It is clear from the above discussions that current and displacement stiffness are important parameters for characterising the performance of magnetic bearing and thus, stiffness measurement is a very essential research topic. This, however, most accounts on such a measurement method were limited at 0 Hz and have regarded the measured values as constants, which is obviously not feasible for high-speed conditions. To adequately verify the correctness of the proposed dynamic stiffness on this research as well as obtain the actual stiffness values for RAMB with varying rotation frequencies, a dynamic stiffness measurement method is proposed under this section.

In general, stiffness is measured on account of Eq. (19), which in turn, can be transformed to *f* = *k*_*x*_·(*h* − *h*_0_) + *k*_*i*_·*i*. For this expression, *f* represents the electromagnetic force of the measured channel, *h* donates the rotor displacement, *h*_0_ donates the magnetic centre position of RAMB, *i* donates the control current in the RAMB coils and *k*_*x*_ and *k*_*i*_ donate the measurement dynamic current and displacement stiffness, respectively. As the electromagnetic force of RAMBs in MSMP balances the gravity of the rotor with certain frequency, the electromagnetic force of each RAMB in the measured channel can be calculated with the solution *f* = *f(mg)* = *G/2*, with *m* indicating the rotor mass and *G* donating the rotor gravity. Consequently, the control current can be represented as:

26 $$i(\omega )=-\,\frac{{k}_{x}(\omega )}{{k}_{i}(\omega )}(h-{h}_{0})+\frac{f(mg)}{{k}_{i}(\omega )}.$$

Under the assumption that the magnetic centre position *h*_0_ is known, measurement of different displacements near *h*_0_ and the corresponding control currents at any rotation speed will result to the finding the current and displacement stiffness at the rotation speed through Eq. (26). Particularly, the magnetic centre position needs only to be determined under static levitation according to its independence from the rotor speed.

Accordingly, there are two main stages in the proposed measurement method. As in the other methods described in^20–22^, the primary stage is determining the magnetic centre. If say, for the *x*-channel in RAMB the rotor is suspended stably along the *x*+ direction at 0 Hz, one coil current value of the RAMB can be obtained and then by inverting the rotor to stably along the *x-*direction, another coil current value can be obtained. If the suspended position of the rotor is adjusted until the two current values are equal, the rotor position that results could be recorded as the magnetic centre.

The secondary stage involves determining the dynamic current and displacement stiffness, which is much different from the literature^20–22^, by adjustment of the rotor frequency. When the rotor is suspended at the magnetic centre position, the corresponding control current for certain frequency *ω* is recorded as *i*_0_. Under this condition, the rotor is consequently adjusted to a position *h*_*j*_ near *h*_0_ and the corresponding control current is recorded as *i*_*j*_, where *j* = 1…n, that is, *n* equilibrium positions around *h*_0_ are transformed through the adjustment of the reference value of the displacement sensor in the control programme for RAMB. According to Eq. (26), the corresponding current and displacement stiffness for *ω* can be expressed through Eqs. (27) and (28). Apparently, on the basis of the measured *h*_0_, *h*_*j*_, *i*_0_(*ω*) and *i*_*j*_(*ω*), the current and displacement stiffness of RAMB under different frequency can be obtained via changing the rotor speed and repeating the steps.

27 $${k}_{i}(\omega )=\frac{f(mg)}{{i}_{0}(\omega )},$$

28 $${k}_{x}(\omega )=-\,\frac{1}{n}\mathop{\sum }\limits_{j=1}^{n}{k}_{i}(\omega )\cdot \left(\frac{ij(\omega )-{i}_{0}(\omega )}{{h}_{j}-{h}_{0}}\right).$$

### Measurement and experiments

**Figure 15:**
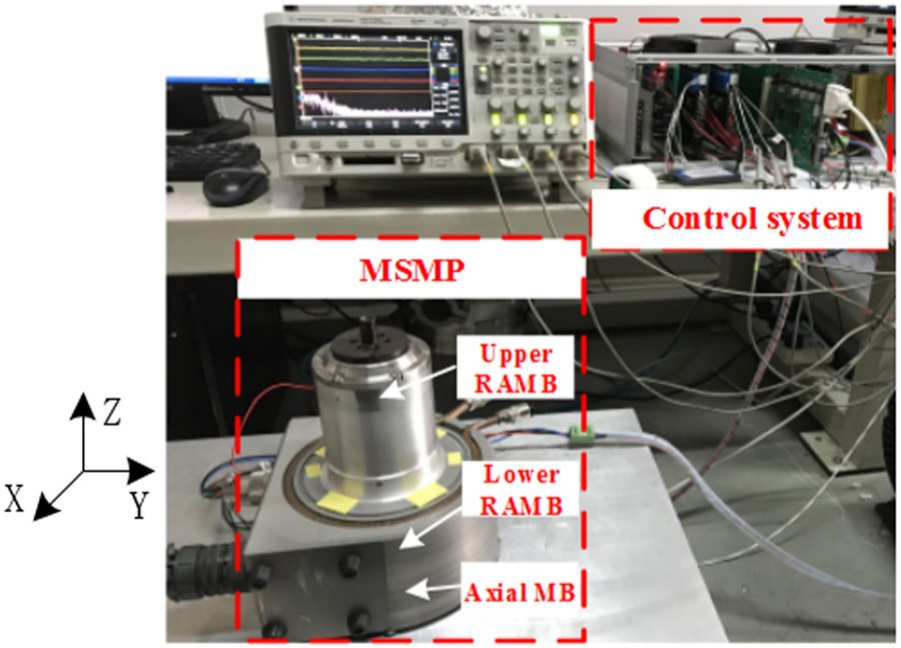
Measurement test system.

**Figure 16:**
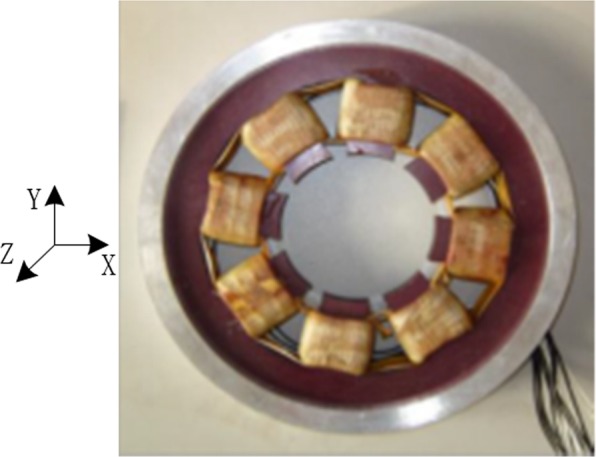
The RAMB prototype.

**Figure 17:**
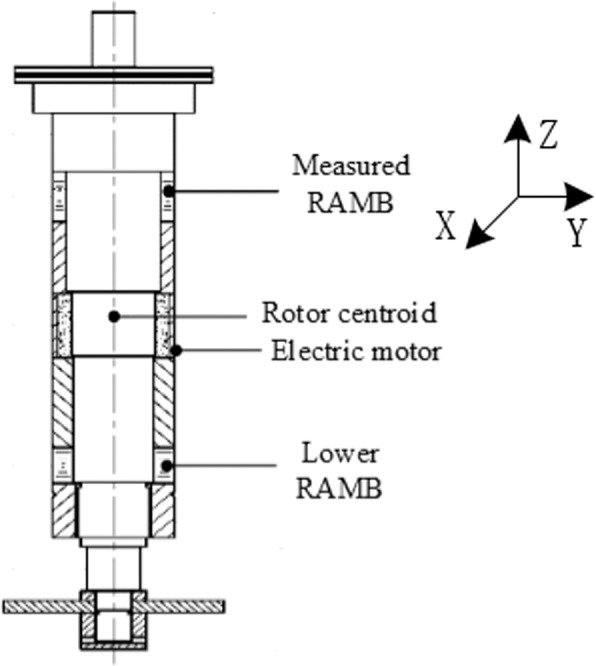
Rotor structure.

The feasibility of the proposed dynamic stiffness characteristics and measurement method, the dynamic stiffness test experiment of RAMB was completed in a MSMP platform with a rated speed of 500 Hz (30000 r/min), as illustrated in Fig. 15. The MSMP model is as shown in Fig. 1 and the measured RAMB prototype and the centre of rotor mass are as depicted in Figs. 16 and 17, respectively.

**Figure 18:**
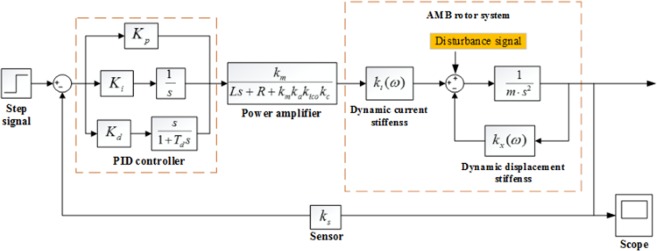
Model of the RAMB control system.

Figure 18 shows the principle block diagram of the RAMB control system, in which the control algorithm adopted in this work is an incomplete differential PID control strategy whose transfer function is reflected in Eq. (25). Upon application of the proposed dynamic stiffness model in the control model, the power amplifier can transform the output voltage signal of the controller into the current signal of the coil, in order to produce the required electromagnetic force. Table 2 provides a list of the main parameters. For the RAMB control system, the rotor displacement is directly measured with a displacement sensor and then compared to the reference signal of displacement set in the programme, after which the PID operation is performed so as to control the rotor to suspend stably at the reference position. Moreover, the reference signal can be regulated online via the control programme, which means that at a certain frequency, the change of rotor position can be achieved by adjusting the displacement reference signal online. Practically speaking, the measurement of dynamic stiffness for RAMB would be convenient and efficient with utilisation of the proposed measurement method.

**Figure 19:**
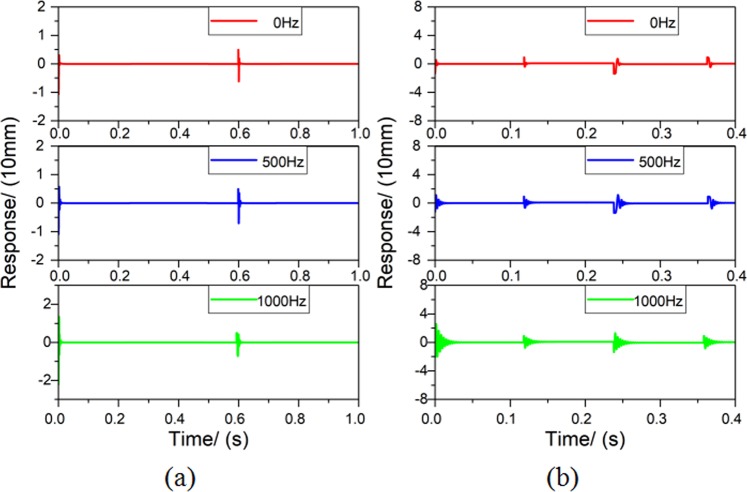
Simulation results for a RAMB system (**a**) with unit impulse and (**b**) with white noise.

During the experiment process, some factors as the interference caused by rotor unbalance will influence the performance of the RAMB system. To better simulate the system, two noise signals including a unit impulse signal and a white noise signal that result in a signal-to-noise ratio of 14 dB, were introduced for the simulation conducted in Matlab/Simulink. Table 3 provides the control parameters utilised while the representative simulation results obtained by online tuning are shown in Fig. 19. Both indicate that the RAMB system with the chosen control parameters can remain stable and is less affected by rotor unbalance and noise.

**Table 3:** Parameters of the PID controller.

Parameter	Value	Parameter	Value
Integral coefficient(*K*_*i*_)	0.049	Proportion coefficient (*K*_*p*_)	1.00
Differential time constant (*T*_*d*_)	0.0005	Differential coefficient (*K*_*d*_)	0.01

#### Dynamic current measurement and results

In the experiment, the magnetic centre was determined according to the primary stage described in the proposed measurement method, with the bias current set at 0.38 A. As a consequence, the control current can be measured when the rotor rotates at the magnetic centre of RAMB with different frequencies and the corresponding dynamic current stiffness under each speed can be obtained through force analysis of the MSMP system and by using Eq. (27). The relationship between the measured dynamic current stiffness and rotor frequency relative to the analytical and FEM results is depicted in Fig. 12.

**Figure 20:**
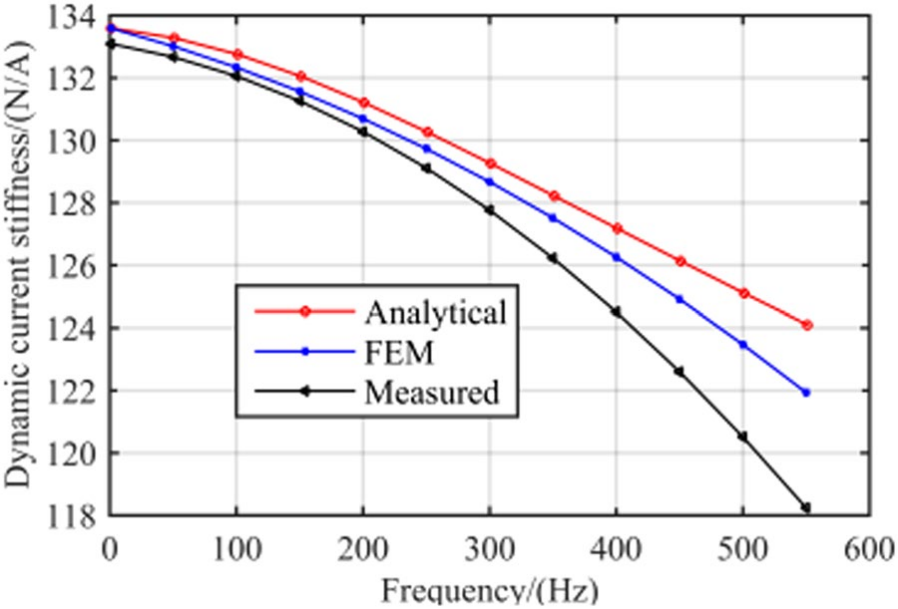
Comparison between the measured dynamic current stiffness at different frequencies and the analytical and FEM results.

By adoption of the measurement method presented in literature^20–22^, the RAMB current stiffness can be obtained in the measurement test system described herein as 133.6 N/A, with no variation in the rotor frequency. Nonetheless, Fig. 20 clearly emphasises that the current stiffness measured by the proposed method would change with rotor speed, with the RAMB current stiffness in the rated speed of 500 Hz being 120.5 N/A. Moreover, comparing the measured values with the analytical values and FEM results, the maximum error between the measured values and analytical values was 5.0% (at 550 Hz) whereas the maximum error between the measured results and FEM results was 3.1% (at 550 Hz). This indicated the good agreement between the results of the experiment and those in both the analytical analysis and FEM results.

#### Dynamic displacement stiffness measurement and results

In order to obtain a convenient measurement of the dynamic displacement stiffness, the attitude of the prototype of the measured MSMP was adjusted so that the *x*-axis faces upward in the vertical direction and the magnetic centre of RAMB was set as the coordinate origin. Under such condition, the magnetic force in the RAMB would be zero when the rotor is suspended stably, such that the control current is *i*_0_ = 0 with *h*_0_ = *0*. Consequently, Eq. (28) can be inverted into (29) yielding

29 $${k}_{x}(\omega )=-\,\frac{1}{n}\mathop{\sum }\limits_{j=1}^{n}{k}_{i}(\omega )\cdot \frac{{i}_{1}(\omega )}{{h}_{j}}.$$

**Figure 21:**
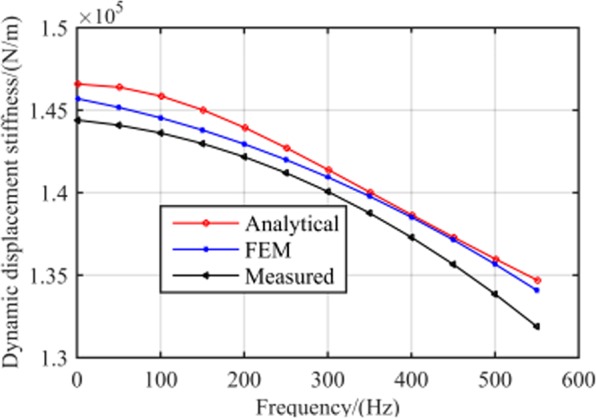
Measured dynamic current stiffness at different frequency compared with analytical results and FEM results.

Finally, the dynamic displacement stiffness can be obtained by the recorded control current values via a series of set equilibrium positions under different rotor frequencies in accordance to Eq. (29). Figure 21 shows a comparison between the measured dynamic displacement stiffness against rotor frequency and the analytical and FEM results.

By adopting of the measurement method presented in literature^20–22^, the RAMB displacement stiffness obtained through the proposed measurement test system was 1.44 × 10^5^ N/m, which is regarded as a constant and is unrelated with the rotor frequency. Nonetheless, it is discerned that the displacement stiffness of RAMB obtained using the proposed measurement method varied with rotor frequency, where the displacement stiffness of RAMB in the rated speed of 500 Hz was 1.34 × 10^5^ N/m. Furthermore, the experimental results were consistent with the analytical data and FEM results and produced low maximum errors of 2.3% at 550 Hz and 1.5% at 550 Hz.

Figures 20 and 21 illustrate that the variation trends as rotor frequency increases for theoretical analysis, FEM and the measured results were consistent. Compared to the analytical and FEM results, the maximum error of measurement was 5.0% and 2.3%, respectively, for current stiffness and displacement stiffness, which is considerably within the acceptable range. This result supports that the proposed dynamic stiffness model and measurement method can perform well and is extremely significant for improving the dynamic performance of RAMB. Additionally, the errors for the three curves in Figs. 20 and 21 could be attributed to two aspects, namely:

- The experiments were performed on a prototyped MSMP, which suggests that interference from the electric motor was unavoidable. To describe the scenario, the electric motor’s position in Fig. 16 was quite close to the measured RAMB, by which the electromagnetic field generated by RAMB is expected to be superimposed with the electromagnetic field of the motor. Additionally, with high rotation speed, the motor would have a complex influence on RAMB for a varying magnetic field under eddy current effects.
- Some ideal hypotheses were aimed to promote calculation convenience in the theoretical analysis part, say, the laminated material of the rotor was linear and the magnetic flux was a spatial function varying with time in the Z-direction.

The errors between the measured results and theoretical analysis can be minimise in future works by focusing on the separation design of the electric motor and measured RAMB, for elimination of the influence generated by the motor.

## Discussion

The content of this work highlighted the proposal of an accurate model of dynamic stiffness, along with a measurement method of dynamic stiffness for RAMB. It conducted an investigation of dynamic stiffness characteristics influenced by rotor frequency for RAMB by application of the dynamic magnetic circuit method and FEM. Based on the results, dynamic stiffness exhibited remarkable variation with an increase in frequency. The proposed measurement method is a two-stage process. The magnetic centre is first determined so that the dynamic stiffness under a certain frequency can be obtained through measurement of different current values when the rotor is adjusted to different equilibrium positions near the magnetic centre. The method was validated by an experiment on a prototyped MSMP. Upon comparison of the experimental values to the theoretical results and the values obtained by FEM, as the rotor rotates at the same frequency, the maximum error of current stiffness was 5.0% and 3.1%, respectively, while the maximum error of the displacement stiffness was 2.3% and 1.5%.

The findings herein have shown great progress in the research of the dynamic properties of RAMB and have as well provided an evaluation standard for dynamic performance improvement and structure optimisation of RAMB.

## Method

### Theoretical analysis for dynamic stiffness

The theoretical analysis for the dynamic stiffness model was based on the RAMB structure in MSMP, as shown in Fig. 1. The corresponding dimensions are described in Table 1 in the main text.

### FEM analysis for dynamic stiffness

The parameters in the FEM magnetic bearing model were consistent with the parameters in Table 1. The 2D FEM mesh model of RAMB in Fig. 2 was obtained as Fig. 12 in the main text, in which the plane53 magnetic structure unit was used for a perfect performance of transient magnetic field analysis. In addition, in the model the rotor part was meshed by mapped grid mode while the remaining part was meshed by free mode. In particular, the areas of air gap between the rotor surface and the fixed rotor, where the electromagnetic field exhibits such a great variation, were finely meshed. In the FEM simulations, the bias current was set at 0.38 A, which is expected to generate a corresponding bias magnetic flux density. The rotor was set with different rotor frequency values from 0 to 800 Hz, in the ANSYS software, followed by FEM simulation for each respective speed.

### Dynamic stiffness measurement and experiments

The experimental conditions and control system are shown in Fig. 15 through 18. The main coefficients are defined in Tables 2 and 3 in the main text.
